# Multi-label deep learning for comprehensive optic nerve head segmentation through data of fundus images

**DOI:** 10.1016/j.heliyon.2024.e36996

**Published:** 2024-09-01

**Authors:** Najdavan A. Kako, Adnan M. Abdulazeez, Diler N. Abdulqader

**Affiliations:** a Department of Information Technology, Technical College of Duhok, Duhok Polytechnic University, Duhok, Kurdistan Region, Iraq; b Department of Energy Engineering, Technical College of Engineering, Duhok Polytechnic University, Duhok, Kurdistan Region, Iraq; c Department of Computer and Communications Engineering, Nawroz University, Duhok, Kurdistan Region, Iraq

**Keywords:** Blood vessels, HRF dataset, Optic disc and cup, Peripapillary atrophy zones, Retinal fundus images, Semantic segmentation

## Abstract

Early diagnosis and continuous monitoring of patients with eye diseases are critical in computer-aided detection (CAD) techniques. Semantic segmentation, a key component in computer vision, enables pixel-level classification and provides detailed information about objects within images. In this study, we present three U-Net models designed for multi-class semantic segmentation, leveraging the U-Net architecture with transfer learning. To generate ground truth for the HRF dataset, we combine two U-Net models, namely MSU-Net and BU-Net, to predict probability maps for the optic disc and cup regions. Binary masks are then derived from these probability maps to extract the optic disc and cup regions from retinal images. The dataset used in this study includes pre-existing blood vessels and manually annotated peripapillary atrophy zones (alpha and beta) provided by expert ophthalmologists. This comprehensive dataset, integrating existing blood vessels and expert-marked peripapillary atrophy zones, fulfills the study's objectives. The effectiveness of the proposed approach is validated by training nine pre-trained models on the HRF dataset comprising 45 retinal images, successfully segmenting the optic disc, cup, blood vessels, and peripapillary atrophy zones (alpha and beta). The results demonstrate 87.7 % pixel accuracy, 87 % Intersection over Union (IoU), 86.9 % F1 Score, 85 % mean IoU (mIoU), and 15 % model loss, significantly contributing to the early diagnosis and monitoring of glaucoma and optic nerve disorders.

## 1 Introduction

In 2022, the World Health Organization estimated that 253 million people are blind, visually impaired, or have low vision, with glaucoma being the second leading cause of blindness globally [1]. Glaucoma is a complex eye condition characterized by optic nerve damage due to increased intraocular pressure from fluid accumulation [2]. It can be of two types: open-angle and closed-angle. To manage the large volume of fundus images and aid in diagnosis, integrating digital image processing algorithms has become crucial. These algorithms offer automated and efficient solutions for analyzing and interpreting fundus images, assisting ophthalmologists in detecting and diagnosing eye disorders effectively [3,4].

The advancement of computing technology has allowed clinicians to handle more patients and improve diagnostic accuracy. Despite this, the manual inspection of retinal images to identify and monitor eye diseases remains common, which is time-consuming and relies heavily on physician expertise. Some conditions may require lengthy examinations over several years for accurate detection and treatment [5,6].

In the field of medical imaging tools, several solutions have been developed to identify eye diseases and related disorders [7]. Some tools are specifically designed to detect changes between pairs of retinal images, aiding experts in monitoring disease indicators over time. However, practical scenarios can introduce deformations in the sequence of fundus images, making image alignment challenging. Moreover, retinal images may contain texture-less regions and uneven illumination, further complicating accurate matching of anatomical features [8]. Optic disc and cup segmentation is a crucial step in analyzing and monitoring eye diseases like glaucoma. It helps detect and assess the enlargement of the optic cup, indicating glaucoma when the cup-to-disc ratio (CDR) is larger than 0.3. Various segmentation methods, including manual, semi-automatic, and automatic approaches, can be used based on the application. Optic disc and cup segmentation offer early diagnosis, timely treatment, disease progression monitoring, and aid in research for new treatments [9].

Segmenting blood vessels in eye diseases is crucial for early diagnosis, monitoring disease progression, and research. It helps identify early signs of diseases like diabetic retinopathy and age-related macular degeneration, guiding prompt treatment. Various segmentation methods - manual, semi-automatic, and automatic - can be employed [10].

Similarly, segmenting peripapillary atrophy (PPA) alpha and beta zones is essential in eye diseases. Early PPA diagnosis identifies individuals at risk, facilitating early treatment. Segmentation aids disease monitoring and researching causes and treatments. Manual, semi-automatic, and automatic segmentation methods are used based on the application. PPA zones differ in RPE loss and retina thinning. Zone size evaluation assesses glaucoma severity and progression. A larger alpha zone or a significant discrepancy between the alpha and beta zones of peripapillary atrophy is a sign of advanced glaucoma. This is because the alpha zone is the outermost zone of peripapillary atrophy, and it is typically the first zone to be affected by glaucoma. As glaucoma progresses, the beta zone may also become affected [11].

**Fig. 1:**
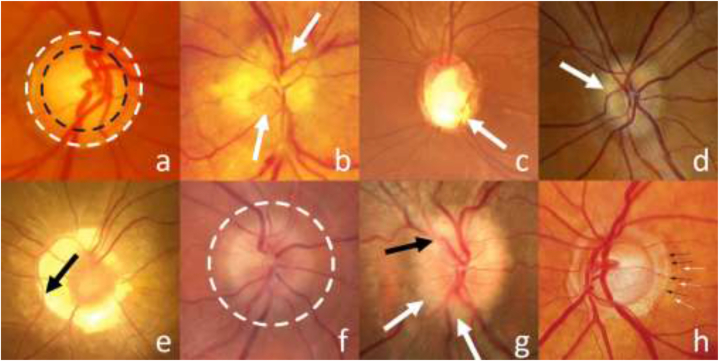
Typical optic nerve diseases in color fundus images. This figure shows patches of color fundus images that are affected by different optic nerve diseases. The diseases are: Glaucoma (a), Anterior ischemic optic neuropathy (AION) (b), Optic nerve drusen (d), Optic nerve pit (e), Optic neuritis (f), Papilledema (g), and PPA β zone (black arrows) - PPA α zone (white arrows) (h).

Optic nerve disorders present a significant obstacle due to their diversity and complexity, as shown in Fig. 1. Fundus images can display various diseases, each with distinct characteristics in shape, color, size, and position. This diversity makes it difficult to develop a comprehensive set of features covering all categories of optic disorders. Additionally, variations in morphological elements like the optic disc, cup, blood vessels, and peripapillary atrophy (PPA) among individuals pose another critical challenge. Designing an automated detection system becomes complicated as it needs to accurately differentiate between anatomical structures and general optic nerve disorders, rather than focusing solely on specific conditions. Thus, detecting and localizing anomalies associated with unknown types of these conditions from fundus images remain challenging tasks in current research efforts.

Overall, Segmentation of optic disc and cup, blood vessels, and PPA alpha and beta zones is essential for early diagnosis, monitoring, and research of ocular diseases like glaucoma. Manual segmentation is labor-intensive and time-consuming, driving the need for automated methods. Semantic segmentation, a computer vision task, assigns categorical labels to pixels in an image. These segmentation techniques aid in timely treatment, treatment plan adjustments, and new treatment development, ultimately enhancing patient outcomes.

### 1.1 Related work

Existing techniques for segmenting ocular structures of the optic nerve can be divided into three categories: blood vessels, optic disc and cup, and peripapillary atrophy zones. These techniques involve segmentation, classification, and segmentation followed by classification for various multi-tasks.

The first group focuses on semantic segmentation of retinal blood vessels using various classical and traditional methods such as Canny edge operators [12], gradient-based edge operators [13], Sobel based edge operators [14], etc., as well as supervised methods like GMM [15]. These techniques often require expert knowledge, manual feature extraction, and mathematical operations, leading to inefficiency and unreliability. To overcome these limitations, deep learning approaches have been proposed, although they demand large-scale training data and computational resources.

The second group aims to segment the optic disc and cup in retinal images. Level set-based approaches [16] are versatile but time-consuming and may lead to under- or over-segmentation. Threshold-based approaches [17] are fast and simple but may not be accurate in regions with flat and broad pixel value ranges. Clustering-based approaches [18] are effective in noise removal and differentiation between homogeneous and heterogeneous regions. Machine learning [19] and deep learning techniques [20] have been employed, showing promising outcomes in improving accuracy and efficiency.

The third group focuses on segmenting peripapillary atrophy, particularly the alpha and beta zones. Studies have used various approaches, including scanning filters, thresholding, and a modified Chan–Vese model. Deep learning has also been utilized for automatic detection of PPA, enabling the concurrent calculation of disc and PPA-disc areas. While manually designed features used in previous studies are effective, they can be time-consuming and may oversimplify the problem. Therefore, researchers have explored conventional medical image processing and computer vision approaches to identify PPA in retinal images [21].

Recent advancements in retinal layer segmentation, particularly focusing on finer details within the retina, have gained attention. Among the employed configurations, the Attention U-Net model with ResNet50 as the encoder backbone exhibited the highest accuracy of 99.53 % in segmenting the Optic Disc using the RIM-ONE dataset [22]. This research addresses critical challenges in glaucoma detection, demonstrating significant improvements in sensitivity (95.2 %) and specificity (97.5 %) compared to existing methods [23]. This improved segmentation accuracy could be valuable for earlier diagnosis of various retinal diseases. The authors propose a simplified U-Net architecture for fast segmentation of the optic disc and retinal vessels in glaucoma detection [24]. Additionally, another study proposes MSA-UNet, a U-Net architecture with multi-scale convolutions and attention gates for precise segmentation of the optic disc and cup in retinal fundus images [25].

Beyond segmentation, classifying retinal images according to disease states is crucial for clinical diagnosis. The study [26] addressed class-imbalanced issues in the original datasets by performing augmentation techniques, including reshaping, to prevent model overfitting and enhance classification accuracy using different CNN architectures. The authors in Ref. [27] proposes a Computer-Aided Diagnosis (CAD) system using U-Net for optic disc and cup segmentation, followed by logistic regression to classify glaucoma based on the relationship between these features, instead of relying on the Optic Cup to Disc Ratio (CDR) formula. The study [28] utilized the Messidor-2 and BRSET datasets for developing deep learning models to evaluate referable diabetic retinopathy, achieving high accuracy in classifying fundus images and diabetic macular edema. They compared ResNet50V2 and InceptionV3 for diabetic retinopathy classification on fundus images, achieving an accuracy of 80 % with ResNet50V2 [29].

Integrating segmentation with subsequent classification tasks has emerged as a powerful approach for comprehensive image analysis. Prior research by Ref. [30] utilized specialized network architectures like CDED-Net and aggregation channel attention network to enhance the segmentation accuracy and classification of the optic disc and cup. By employing advanced techniques like SLIC and normalized graph cut algorithms for optic disk segmentation, the method in Ref. [31] outperforms basic image processing methods, demonstrating superior performance compared to pre-trained neural networks like VGG19, InceptionV3, and ResNet50V2. By leveraging ensemble-based deep learning models and advanced pre-processing techniques like PCA and CLAHE, the proposed method in Ref. [32] achieves significant improvements in classification accuracy and overcomes complexities associated with traditional approaches. The paper [33] presents a novel deep learning framework utilizing attention-based Swin U-Net for accurate segmentation of fundus images, followed by a hybrid deep learning model for diabetic retinopathy classification.

### 1.2 Contributions

In this paper, we propose a method for multiclass semantic segmentation utilizing the U-Net architecture with transfer learning to fully segment the optic disc and cup, blood vessels, and PPA alpha and beta zones in retinal images, which typically exhibit various characteristics. Unlike methods that focus on segmenting specific regions of the fundus image, our approach aims to segment the entire fundus image, particularly the areas in and around the optic disc. The contributions of this work can be summarized as follows.

• First, we developed two U-Net models, BU-Net and MSU-Net, for optic disc and cup segmentation. These models generate probability maps for the optic disc and produce refined binary masks. The segmentation process includes polar transformation of the region of interest, followed by MSU-Net prediction and image processing to enhance mask accuracy.
• Second, we created a ground truth for the HRF dataset, which includes the optic disc, retinal vessels, and alpha/beta zone peripapillary atrophy. The BU-Net and MSU-Net models delineate optic disc boundaries, retinal vessels are directly extracted from the dataset, and alpha/beta zones are manually annotated. This establishes a robust ground truth for accurate analysis of the HRF dataset.
• Third, we present a comprehensive framework for semantic segmentation using U-Net, both from scratch and with transfer learning. Data preprocessing normalizes the training and mask datasets, enabling effective multiclass segmentation. The flexible U-Net architecture handles various segmentation tasks, and the integration of transfer learning with pre-trained models potentially enhances performance.

The paper is structured as follows: Section IIprovides a detailed explanation of the proposed methodology, including the underlying principles and the employed algorithm. Section IIIpresents the experimental results obtained by applying the proposed methodology, including performance metrics, comparisons with existing techniques, and discussions of the findings. The results are analyzed and interpreted to offer insights into the effectiveness of the proposed method. Finally, concluding observations are presented in Section IV.

## 2 Proposed model

**Fig. 2:**
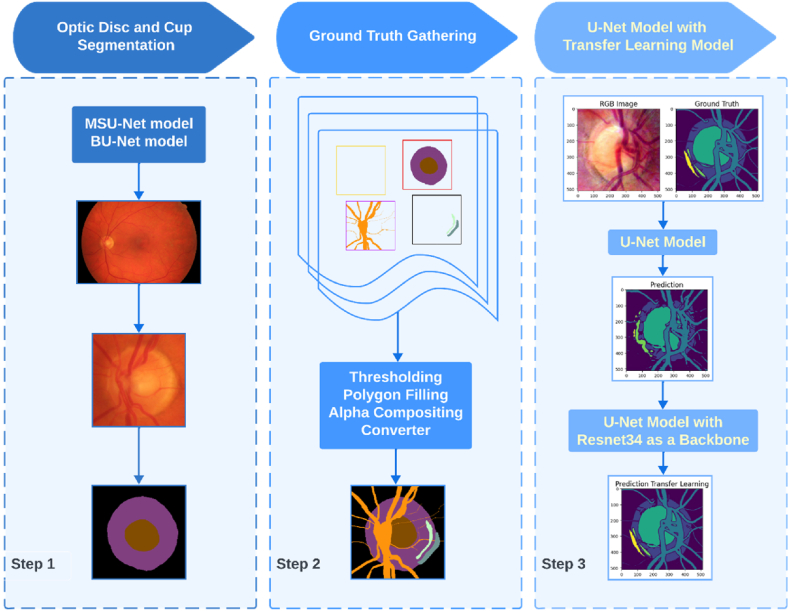
The main flowchart of proposed method.

**Fig. 3:**
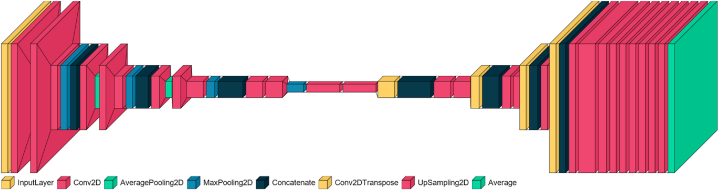
Architecture of MSU-Net.

The proposed model is structured around a meticulously crafted tripartite framework, consisting of three key components: first, the precise segmentation of the optic disc and cup regions; second, the systematic creation of a refined ground truth dataset, which is crucial for model training and validation; and third, the implementation of an intricately designed U-Net architecture, enhanced by transfer learning techniques, to facilitate the complex multiclass classification process. The main flowchart of the method, illustrated in Fig. 2, provides an overview of the approach. Before delving into the core methodology, certain preprocessing steps are introduced to ensure that the necessary preparations are made. These preprocessing phases are essential for optimizing the performance and accuracy of the segmentation algorithm (see Fig. 3).

### 2.1 Preprocessing

In a clinical setting, retinal images may exhibit variations in scale, color, and morphological elements due to individual differences. To address these challenges, a series of preprocessing steps using Python standardizes and enhances the images, ensuring robust and accurate segmentation. The preprocessing phases involve the following steps.

1) Resizing the original input image to 512 × 512 pixels to match the input size required by the deep learning model.
2) Adding an extra dimension to the resized image and scaling it by 255 to prepare it for model processing.
3) Converting the region of interest (ROI) around the optic disc to polar coordinates and rotating it by −90° to fit the model's required format.
4) Generating a binary mask for segmentation, normalizing pixel values, and scaling them to a range of 0–255. The result is then converted to an unsigned 8-bit integer format.
5) Modifying specific pixel values based on certain conditions to enhance the visual representation of the segmented result.

The blood vessels, PPA alpha, and beta zones were manually marked by experts and extracted using a thresholding method. The interiors of these objects are filled using a polygon filling technique. Once the objects are individually extracted, they are combined using the alpha composite method. Finally, the images are converted from RGBA mode to RGB mode using the convert method. RGBA mode includes an additional channel for transparency (alpha) information, whereas RGB mode does not. By converting the image to RGB mode, the alpha channel is discarded, resulting in a standard RGB image, as depicted in Fig. 2.

The following equations summarize the operations involved in thresholding by contour, polygon filling, alpha composite, and converting from RGBA to RGB

(1) Threshold=Mean+k×Standarddeviation

(2) $$Mean=\frac{\sum Pixel\;Value}{No.\;of\;Pixels}$$

(3) $$Standard\;deviation={\frac{\left(\sum Pixel\;Value-Mean\right)}{No.\;of\;Pixels}}^{2}$$

where k is a constant that is typically between 1 and 3.

The equation for polygon filling is:

(4) $$\begin{array}{l} for\;each\;pixel\;p\;in\;polygon\;P\text{:}\\ if\;p\;is\;inside\;P\text{:}\\ p=foreground\\ else\text{:}\\ p=background \end{array}$$

The equation for alpha composite is:

(5) inputimg=(sourceimg×α)+(destinationimg×(1−α))

where α:is the alpha value of the source image, img: is an image.

The equation for converter (RGBA to RGB) is:

(6) RGB=(1−α)×Background+α×Color

The resize function is:

(7) $$I_{new}\left(x,y\right)=InterpolationMethod\left(I_{input},x,y,Order,Mode,Value_{extrapolated}\right)$$

Let's denote I_input_: as the input image, I_output_: as the output image, Order: as the order of the interpolation method (e.g., linear, cubic, etc.), Mode: as the mode of interpolation (e.g., nearest neighbor, bilinear, etc.), Value_extrapolated_: as the value to use for pixels that are extrapolated and (x,y): represent the coordinates of the pixel.

The final steps of preprocessing are performed before training the semantic segmentation model. Firstly, the training and mask images are imported, where the images are read, resized to 512 × 512, and converted to NumPy arrays. Next, the pixel values of the training images are normalized using Min-Max scaling. The mask images are assigned class labels based on a label dictionary, and the resulting label images are obtained. The dataset is then split into training and testing sets using a train-test split of 80:20. Finally, the data is ready for training the U-Net model for semantic segmentation.

### 2.2 Optic disc and cup segmentation

The overall diagram depicting the optic nerve head and cup segmentation approach is presented in Fig. 6. The methodology incorporates the utilization of a U-Net deep network and a polar transformation of fundus images. The U-Net architecture is a comprehensive multi-label deep network that comprises four key components.

• Multi-scale layer: This layer constructs an image pyramid input and facilitates the fusion of receptive fields at multiple levels.
• MSU-Net: This component serves as the core structure and learns a hierarchical representation.
• BU-Net: This component operates on the initial convolutional layers and provides assist for supervision at deeper layers.
• Multi-label loss function: This function is introduced to ensure the simultaneous segmentation of the optic nerve head and cup regions.
• Polar Transformation

#### 2.2.1 MSU*-*net

The proposed method, "Multi-Scale U-Net for Image Segmentation" (MSU-Net), extends the traditional U-Net design used for medical image segmentation. MSU-Net features an encoder path and a decoder path. In the encoder path, convolutional layers with a filter bank are utilized to generate feature maps, with ReLU activation functions applied. The decoder path also employs convolutional layers to generate feature maps, and skip connections are used to concatenate corresponding feature maps from the encoder with up-sampled feature maps from the decoder. The final layer of the decoder produces a high-dimensional feature representation, which is fed into a trainable multilabel classifier. This classifier employs a 1 × 1 convolutional layer with a Sigmoid activation function to perform pixelwise classification, generating a probability map with K channels (where K = 2 for optic disc and cup). Each pixel in the map is assigned the class with the highest probability. Overall, MSU-Net enhances the traditional U-Net architecture by incorporating efficient skip connections and a multilabel classifier, resulting in accurate class predictions at the pixel level.

#### 2.2.2 Multi-scale input layer

The multi-scale input layer, also known as the image pyramid, is employed in this U-Net implementation to improve segmentation quality. Unlike traditional methods that feed multiple scales of images into separate networks and merge the output maps at the final layer, this approach utilizes a mean pooling layer to down-sample the image within the encoder path, thereby creating a multi-scale input. This method offers several advantages: it integrates multi-scale inputs directly into the decoder layers, preventing parameter bloat and increasing network width in the decoder path. By incorporating the multi-scale input layer, this U-Net implementation enhances the segmentation process by efficiently leveraging information from multiple scales while minimizing unnecessary complexity and computational overhead.

#### 2.2.3 BU-net

The architecture known as "Binary U-Net for Image Segmentation" (BU-Net) incorporates a side output layer that functions as a classifier to generate local maps from the early-stage layers. This side output layer introduces an objective function that combines the side output loss with the final layer loss, using fusion-weight parameters for each side-output layer. By backpropagating the side output loss to the early layers in the decoder path, the side output layer mitigates the issue of gradient vanishing, thereby facilitating the training of early layers. Additionally, the side output layer provides supervision at each scale, improving results through the fusion of multiple scales. The goal function of the side output layer is expressed as follows:

(8) $$L_{S}\left(W,w\right)=\sum_{m=1}^{M}a_{m}L_{s}^{\left(m\right)}\left(W,w^{\left(m\right)}\right)$$

Where.

α_m_ = 0.25 is the loss function.
M: is the side-output number.
W: represents the parameters of all the conventional convolutional layers.
L^(m)^_s_: represents the multi-label loss of the m side-output layer.

#### 2.2.4 Multi-label loss function

The segmentation of the optic disc and cup in this method is approached as a multi-label problem. Unlike traditional segmentation methods that operate within the multiclass framework, where instances are assigned to a single unique label from multiple classes, the multi-label approach employed here trains independent binary classifiers for each class. This approach is well-suited for addressing the overlapping nature of the disc and cup regions. To this end, a multi-label loss function based on the Dice coefficient is proposed. The Dice coefficient serves as a common measure of overlap for evaluating segmentation performance. The multi-label loss function assigns a binary ground truth label to each class (optic disc and optic cup) and incorporates class weights to control the contribution of each class. By accounting for the imbalance between foreground and background regions, the loss function effectively integrates into the back-propagation process using stochastic gradient descent. The defined multi-label loss function, denoted as L_s_, is as follows:

(9) $$L_{S}=1-\sum_{k}^{K}\frac{2w_{k}\sum_{i}^{N}p\left(k,i\right)g\left(k,i\right)}{\sum_{i}^{n}p_{\left(k,i\right)}^{2}+\sum_{i}^{N}g_{\left(k,i\right)}^{2}}$$

Where.

N: is the pixel number
p_(k,i)_ ∈ [0; 1] and g_(k,i)_ ∈ {0; 1}: is predicted probability and binary ground truth label for class k, respectively.
K is the class number.
Σ_k_w_k_ = 1, are the class weights.

Our multi-label loss function, as expressed in Equation (9), is equivalent to the traditional Dice coefficient when K = 1. For our approach, we set K = 2 to perform segmentation of the optic disc and optic cup. It is important to note that the Dice loss function measures the overlap ratio of the foreground mask and addresses the issue of pixel imbalance between the foreground (i.e., optic disc or optic cup) and the background. In our multi-label configuration, a pixel can be independently labeled as either optic disc, optic cup, or both. Consequently, there is no imbalance issue between the optic disc and optic cup. In Equation (9), w_k_ represents the trade-off weight that manages the relative contributions of the optic disc and optic cup. Given the importance of both the optic disc and optic cup in glaucoma screening, we set w_k_ = 0.5. Our multi-label loss function L_s_ is differentiable, yielding the gradient as follows:

(10) $$\frac{\partial L_{s}}{\partial p_{\left(k,i\right)}}=\sum_{k}^{K}2w_{k}\left[-\frac{g_{\left(k,i\right)}}{\sum_{i}^{N}p_{\left(k,i\right)}^{2}+\sum_{i}^{N}g_{\left(k,i\right)}^{2}}+\frac{2p_{\left(k,i\right)}\sum_{i}^{N}p_{\left(k,i\right)}g_{\left(k,i\right)}}{{\left(\sum_{i}^{N}p_{\left(k,i\right)}^{2}+\sum_{i}^{N}g_{\left(k,i\right)}^{2}\right)}^{2}}\right]$$

This loss is efficiently integrated into back-propagation via standard stochastic gradient descent.

By combining the U-Net architecture, multi-scale input layer, side-output layer, and multi-label loss function, this method achieves effective segmentation of the optic disc and cup regions. The localized disc center, polar coordinate transformation, and inverse polar transformation steps are also performed to facilitate the segmentation process.

#### 2.2.5 Polar transformation

In this method, a polar transformation is introduced to enhance the segmentation performance of the optic disc and cup in fundus images. The polar transformation maps each pixel of the original image to a corresponding point in the polar coordinate system based on its radius and angular position relative to the disc center. This transformation imposes spatial constraints, ensuring that the cup is within the disc region, and creates a layered structure that facilitates post-processing. It also provides equivalent data augmentation, allowing for various transformations of the original image within the polar coordinate space. Moreover, the polar transformation addresses bias and overfitting issues by enlarging the cup region through interpolation, balancing the disproportionate cup-to-background distribution.

While a similar polar transformation has been used in previous work for cup detection, our method differs in both motivation and approach. We jointly segment the optic disc and cup regions, considering their mutual relationship within the polar coordinate system. For generating polar coordinates in the analysis of fundus images, 400 distinct bins were utilized, each representing a specific angular segment within the polar coordinate system. This division of the circular range into 400 segments ensures a highly detailed transformation process, providing a comprehensive representation of directional information and facilitating accurate segmentation of the optic disc and cup within the fundus images. The transformation relationship between polar and Cartesian coordinates is as follows:

Polar to Cartesian:

(11) x=r×cos(θ),y=r×sin(θ)

**Fig. 4:**
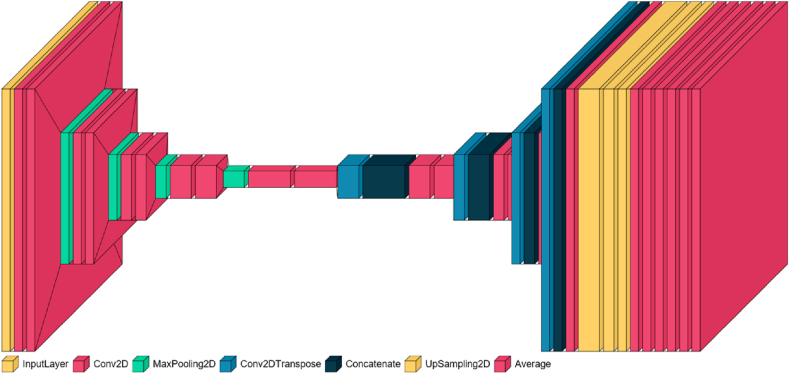
Architecture of BU-Net.

Cartesian to Polar:

(12) r=sqrt(x²+y²),θ=arctan2(y,x)

where r is the distance from the origin, θ is the angle measured from the positive x-axis, x is the Cartesian x-coordinate, and y is the Cartesian y-coordinate (see Fig. 4).

**Fig. 5:**
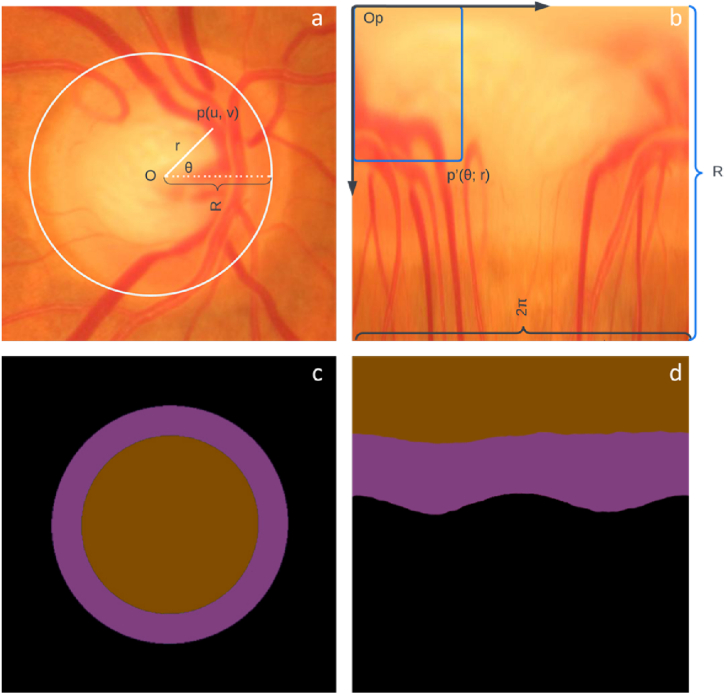
The diagram demonstrates the transformation from Cartesian coordinates (a) to polar coordinates (b) using the polar transformation technique. In Cartesian coordinates, a point p (u; v) corresponds to the point p'(θ; r) in polar coordinates. The ground truth representations (c) and (d) illustrate the respective areas of the optic cup, optic disc, and background, distinguished by the brown, violet, and black regions.

Let us consider the point denoted by *p(u, v)* on the retinal image plane, where the source is defined as the center of the disc *O(u*_*o*_*, v*_*o*_*)*. The Cartesian coordinates *(u, v)* represent the position of the point, as illustrated in Fig. 5a. In the polar coordinate system, the corresponding point is represented as *p'(θ, r)*, as depicted in Fig. 5c. Here, *r* denotes the radius and *θ* represents the directional angle of the original point *p*.

**Fig. 6:**
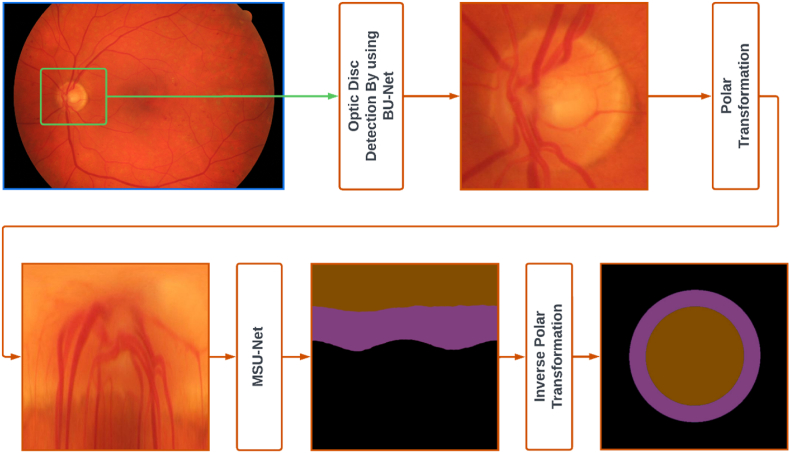
illustrates the overall flowchart of optic disc and optic cup segmentation method.

### 2.3 Ground truth generation for the High-Resolution Fundus (HRF) database

The ground truth for the High-Resolution Fundus (HRF) database was established through a systematic process to accurately identify and annotate specific features within the dataset. The following sections outline the detailed steps involved in creating this ground truth.

Step 1 Optic Disc and Cup Segmentation: The initial step involved segmenting the optic disc and cup from the fundus images. This process utilized advanced computer vision algorithms and techniques, as detailed in Section B. Accurate segmentation of these anatomical structures is essential for further analysis and diagnosis. The optic disc, where the optic nerve enters the eye, and the cup, the central depression within the optic disc, were precisely identified. To validate the segmentation, the MSU-Net model was trained on the ORIGA dataset, which contains ground truth data for the optic disc and cup. This model achieved state-of-the-art results on the ORIGA dataset and demonstrated satisfactory performance in glaucoma screening, as evidenced by the Cup-to-Disc Ratio (CDR) values calculated on both the ORIGA and SCES datasets.

Step 2 Segmentation of Blood Vessels: In the second step, blood vessels within the dataset were segmented. Blood vessel segmentation is crucial for detecting abnormalities such as vessel tortuosity, narrowing, or leakage, which can indicate various retinal diseases. Specialized algorithms were employed to differentiate the vessels from the background and other structures. This step ensured that the ground truth annotations precisely included the location and boundaries of the blood vessels, supporting subsequent analysis and evaluation.

Step 3 PPA Beta and Alpha Zone Marking: The final step involved marking the Peripapillary Atrophy (PPA) beta and alpha zones. PPA, an area of atrophy around the optic disc, is commonly observed in conditions such as glaucoma. The PPA is categorized into two zones: the beta zone and the alpha zone. An ophthalmologist from Duhok Eye Hospital performed the marking of these zones on the fundus images. Utilizing their expertise in retinal pathology, the ophthalmologist carefully identified and delineated the boundaries of these zones. These expert markings provide the ground truth annotations necessary for future research and analysis related to PPA.

**Fig. 7:**
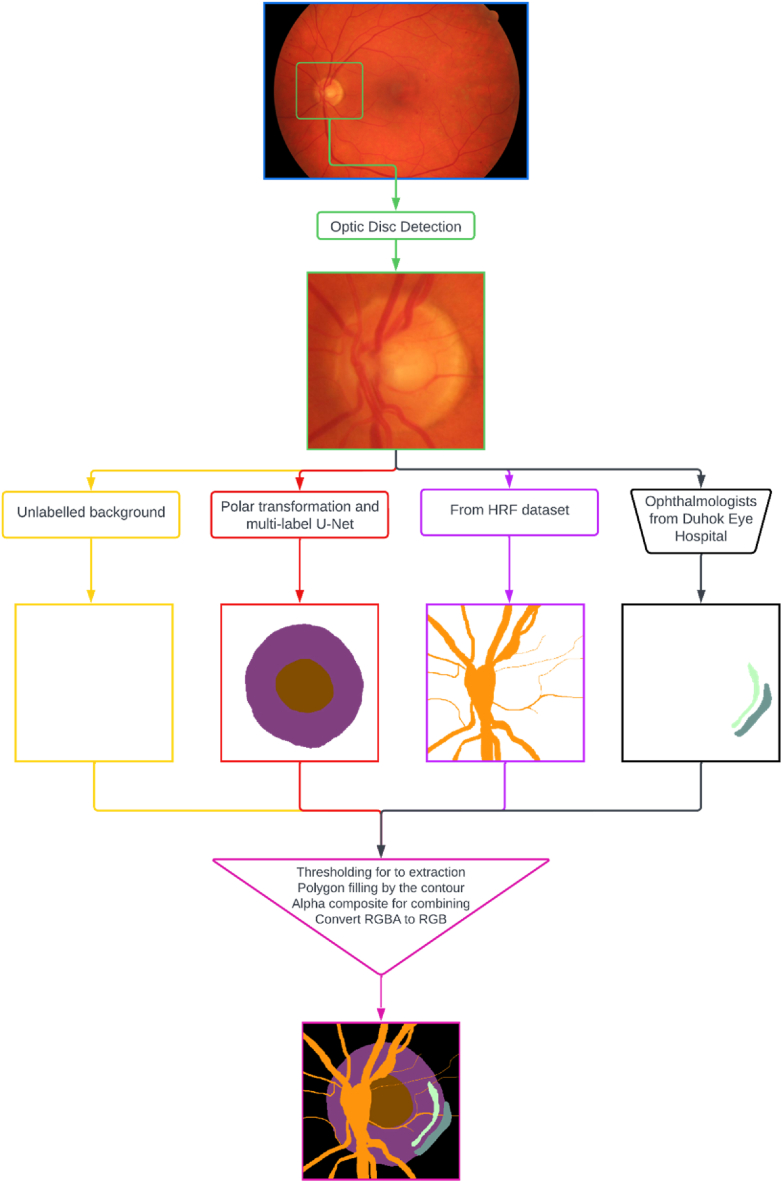
The ground truth establishing of the HRF database.

By following these steps, the ground truth for the HRF database was established, as illustrated in Fig. 7. The combined annotations of the segmented optic disc and cup, blood vessels, and PPA zones offer a valuable resource for tasks such as automated disease diagnosis, image registration, and enhancement algorithms. These annotations serve as a benchmark for comparing and validating results from various computer-based algorithms and methods in retinal image analysis.

### 2.4 Segmentation utilizing the proposed U-Net model

In the realm of bio-medical image segmentation, the U-Net architecture was introduced by Ref. [34]. This architecture operates in two distinct pathways. The first pathway, known as the encoder or contraction path, extracts contextual information from the input image through a series of convolutional layers, dropout layers, and max pooling layers. The second pathway, referred to as the decoder or expanding path, aims to achieve precise localization using transposed convolutions. The U-Net architecture is named for its resemblance to the letter "U," and it is an end-to-end fully convolutional network (FCN), consisting exclusively of convolutional layers without any dense layers. This design allows U-Net to handle images of varying sizes.

In this study, a custom U-Net model was developed for the automatic segmentation of ocular structures in optic nerve images using the HRF dataset. The U-Net architecture was designed from scratch, with the number of layers determined through extensive experimentation. Various layer combinations were tested to identify the optimal configuration for achieving the highest segmentation performance for ocular structures.

**Fig. 8:**
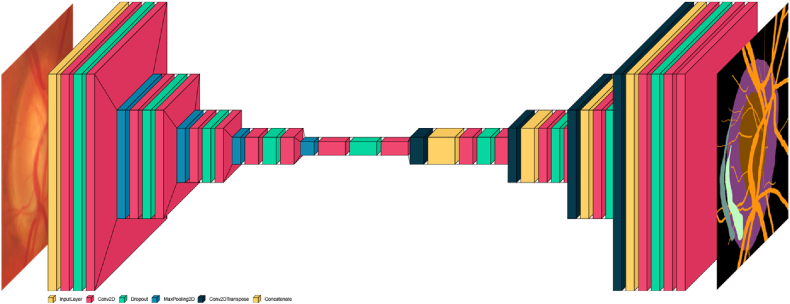
The block diagram of U-Net.

**Fig. 9:**
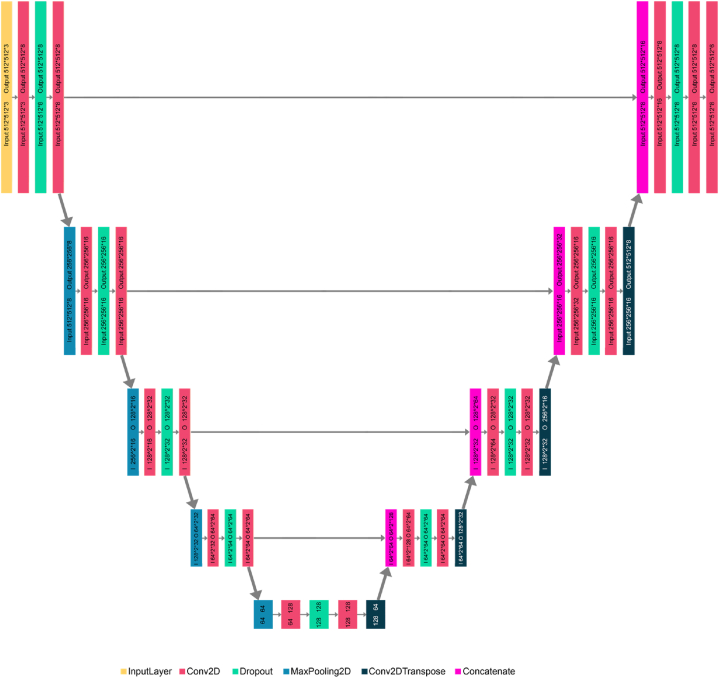
The block diagram illustrates the various layers in the proposed U-Net architecture.

The proposed U-Net model integrates convolutional, dropout, and max-pooling layers. Fig. 8, Fig. 9 illustrate the block diagrams showcasing the layers used in this custom U-Net model. Similar to the standard U-Net architecture, the proposed model consists of two branches: the encoder on the left and the decoder on the right, forming the "U" shape.

Notably, the proposed U-Net model does not include dense layers; instead, it comprises convolutional, dropout, max-pooling, and transposed convolution layers. Additionally, this study employs an input image size of 512 × 512 pixels, differing from the standard U-Net model's image size.

The model overview of the custom U-Net architecture is detailed in Table 1. This table outlines the following attributes for each layer: number of filters, input size, filter size, output size, and number of parameters. Each convolution layer uses a filter size of 3 × 3, with the number of filters doubling at each block, starting from 8 and increasing to 128. The center block includes 128 filters for two convolution layers and 64 filters for one transposed convolution layer. Together, the four down-sampling blocks and the center block constitute the encoder branch. After processing through the encoder branch, the image size is reduced to 64 × 64 × 64.

**Table 1:** Layers outline of the designed U-Net mode.

Block Name	Layer Name	Size of Input Image	Size of Filter	No. of Filters	Activation Function	Size of Output Image	No. of Parameters
Down-sampling 1	InputLayer	512*512*3				512*512*3	0
	Conv2D	512*512*3	3*3	8	ReLU	512*512*8	224
	Dropout	512*512*8				512*512*8	0
	Conv2D	512*512*8	3*3	8	ReLU	512*512*8	284
Down-sampling 2	MaxPooling2D	512*512*8				256*256*8	0
	Conv2D	256*256*8	3*3	16	ReLU	256*256*16	1168
	Dropout	256*256*16				256*256*16	0
	Conv2D	256*256*16	3*3	16	ReLU	256*256*16	2320
Down-sampling 3	MaxPooling2D	256*256*16				128*128*16	0
	Conv2D	128*128*16	3*3	32	ReLU	128*128*32	4640
	Dropout	128*128*32				128*128*32	0
	Conv2D	128*128*32	3*3	32	ReLU	128*128*32	9248
Down-sampling 3	MaxPooling2D	128*128*32				64*64*32	0
	Conv2D	64*64*32	3*3	64	ReLU	64*64*64	18496
	Dropout	64*64*64				64*64*64	0
	Conv2D	64*64*64	3*3	64	ReLU	64*64*64	36928
Centre Block	MaxPooling2D	64*64*64				32*32*64	0
	Conv2D	32*32*64	3*3	128	ReLU	32*32*128	73856
	Dropout	32*32*128				32*32*128	0
	Conv2D	32*32*128	3*3	128	ReLU	32*32*128	147584
	Conv2DTranspose	32*32*128	2*2	64	Linear	32*32*64	32832
Up-sampling 1	Concatenate	64*64*64				64*64*128	0
	Conv2D	64*64*128	3*3	64	ReLU	64*64*64	73792
	Dropout	64*64*64				64*64*64	0
	Conv2D	64*64*64	3*3	64	ReLU	64*64*64	36928
	Conv2DTranspose	64*64*64	2*2	32	Linear	128*128*32	8224
Up-sampling 2	Concatenate	128*128*32				128*128*64	0
	Conv2D	128*128*64	3*3	32	ReLU	128*128*32	18464
	Dropout	128*128*32				128*128*32	0
	Conv2D	128*128*32	3*3	32	ReLU	128*128*32	9248
	Conv2DTranspose	128*128*32	2*2	16	Linear	256*256*16	2064
Up-sampling 3	Concatenate	256*256*16				256*256*32	0
	Conv2D	256*256*32	3*3	16	ReLU	256*256*16	4624
	Dropout	256*256*16				256*256*16	0
	Conv2D	256*256*16	3*3	16	ReLU	256*256*16	2320
	Conv2DTranspose	256*256*16	2*2	8	Linear	512*512*8	520
Up-sampling 4	Concatenate	512*512*8				512*512*16	0
	Conv2D	512*512*16	3*3	8	ReLU	512*512*8	1160
	Dropout	512*512*8				512*512*8	0
	Conv2D	512*512*8	3*3	8	ReLU	512*512*8	584
	Conv2D	512*512*8	1*1	6	Softmax	512*512*6	54

The decoder branch of the model integrates transposed convolution, dropout, concatenation, and convolution layers. The encoder pathway decreases the image size, while the decoder branch enlarges it. It consists of four up-sampling blocks, where the image size increases and the filter count decreases progressively from 128, 64, 32, 16, 8, to 6. A final convolution layer further processes the image, resulting in an output size of 512 × 512 × 8. The input image to the encoder pathway is 64 × 64 × 64, and after passing through all the up-sampling blocks, the output image size is 512 × 512 × 8. Although the output image size matches the input image size, the U-Net model generates a segmented image at its output. This segmented image includes a mask with six objects: optic disc, optic cup, blood vessels, PPA beta zone, PPA alpha zone, and background.

### 2.5 Segmentation utilizing pre-trained transfer learning models

Transfer learning is a deep learning technique where a model trained on one problem is used to solve a related problem. The model is trained on a dataset similar to the target dataset and then adapted by adding new layers for the specific target problem. In this particular research work, the segmentation of ocular structures was carried out by leveraging nine pre-trained transfer learning models: InceptionV3 [35], SeResNet34 [36], VGG19 [37], DenseNet121 [38], InceptionResNetV2 [39], EfficientNetB0 [40], MobileNet [41], and SeResNeXt-50 [36] and ResNet-34 [42]. The U-Net architecture was constructed using several pre-trained models. InceptionV3, an advanced version of InceptionV1, enhances network performance and adaptability with several state-of-the-art convolutional neural network strategies. It offers a deeper network without sacrificing speed compared to its predecessors. SeResNet34, a variant of ResNet with squeeze-and-excitation blocks, achieves a 25 % performance improvement on ImageNet. VGG19, with 19 layers, is effective for image classification, while DenseNet121 increases network depth through dense connections between layers. InceptionResNetV2 combines Inception architecture with residual connections, improving training efficiency and reducing time. EfficientNetB0 utilizes the EfficientNet scaling method for enhanced performance through uniform scaling. MobileNet is optimized for mobile and embedded devices, providing efficient image classification solutions. SeResNeXt-50 merges ResNeXt with squeeze-and-excitation for strong performance, and ResNet-34 with 34 layers addresses degradation problems in deep networks.

Despite their effectiveness in general applications, these eight models exhibited unexpectedly lower performance on the HRF dataset. This can be attributed to several factors. First, the HRF dataset consists of only 45 fundus images, a very small sample size that often leads to overfitting, where models perform well on training data but generalize poorly to new data. The substantial variability in fundus images—such as differences in lighting, contrast, and anatomical features—further challenges model generalization. Imbalances in pathology types or anatomical variations within the dataset also hinder effective learning. Moreover, the domain shift between pre-training datasets and the HRF dataset affects performance. Fundus images differ significantly from the general images used to pre-train networks like DenseNet and EfficientNet. Evaluation metrics such as Pixel Accuracy, IoU, Dice Coefficient, mIoU, and Model Loss are sensitive to dataset characteristics. Segmentation of small and intricate structures is particularly challenging with limited data. Some architectures, like Inception-v3 and MobileNet, are optimized more for classification tasks rather than segmentation, contributing to their lower performance in this context. Training stability, hyperparameter selection, and random initialization effects also impact performance, especially with small datasets.

In contrast, the Proposed U-Net and Proposed U-Net + TL (with transfer learning) showed relatively better performance. Segmentation-specific architectures combined with transfer learning are generally more suited for specialized tasks involving limited data. Table 2 compares these transfer learning models based on the number of layers, parameters, and processing time (see Table 3).

**Table 2:** Comparison of transfer learning models.

Model Name	No. of Layers	Total Parameters	Trainable Parameters	Non-trainable Parameters	Processing Time (Hours)
Inception-v3	48	29,933,830	29,897,414	36,416	12.08
SeResNet34	34	24,618,075	24,600,725	17,350	9.61
VGG-19	19	29,062,694	29,058,662	4032	7.99
DenseNet-121	121	12,145,702	12,060,070	85,632	11.24
InceptionResNetV2	164	62,062,278	61,999,750	62,528	10.41
EfficientNetB0	237	10,116,226	10,072,226	44,000	3.58
MobileNet	27	8,337,062	8,313,190	23,872	3.91
SeResNeXt-50	50	34,594,902	34,524,694	70,208	8.75

**Table 3:** Accuracy of different methods on the DRIVE.

Methods	Accuracy
[43]	93.23 %
[44]	80.24 %
[45]	96.98 %
[46]	95.9 % With Enhancement
[47]	96.66 %
[48]	94.03 %
Ours	97.90 %

## 3 Results and discussion

In this study, we evaluated the proposed U-Net model, developed from scratch, alongside nine pre-trained models: InceptionV3, SeResNet34, VGG19, DenseNet121, InceptionResNetV2, EfficientNetB0, MobileNet, SeResNeXt-50, and ResNet-34. The models were produced using Keras, a high-level API for TensorFlow. Keras is a user-friendly, open-source framework designed for neural networks, compatible with both TensorFlow and Theano, and optimized for accelerating deep neural network computations.

**Fig. 10:**
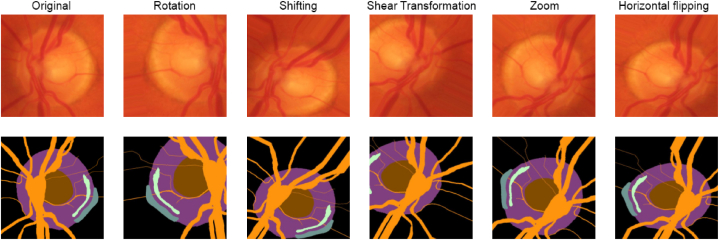
Data augmentation.

All simulations were conducted using Python v3.9.16 in the Jupyter Notebook environment, equipped with TensorFlow and a GPU for efficient processing. Data augmentation techniques, as illustrated in Fig. 10, were applied to the HRF dataset, expanding the training set to 270 images. Despite these efforts, the results did not meet expectations, possibly due to the model overfitting to unrealistic variations introduced during augmentation.

To address these issues, the focus will shift to using a smaller set of high-quality images with augmentations that more accurately reflect natural retinal variations. Additionally, more effective data augmentation techniques will be explored, and the U-Net architecture will be re-evaluated for potential improvements.

### 3.1 Hyperparameter tuning

In this study, the models were configured with the following parameters: a 20 % test size and 80 % training size, a random state of 42, and the Adam optimizer. The loss function used was categorical cross-entropy, with accuracy as the metric. Training was conducted with a batch size of 1, verbosity level 1, and for 100 epochs. The learning rate was set to 0.0001. Activation functions included ReLU for convolutional layers, linear for transposed convolutional layers, and Softmax for the final output layer. Test data and labels were utilized as validation data during the training process.

### 3.2 Evaluation of U-Net

**Fig. 11:**
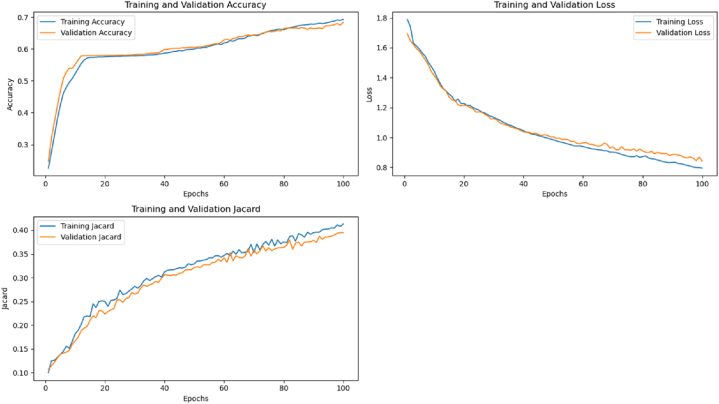
5-Fold cross-validation performance: Comparison of training and validation accuracy, loss, and Jaccard.

**Fig. 12:**
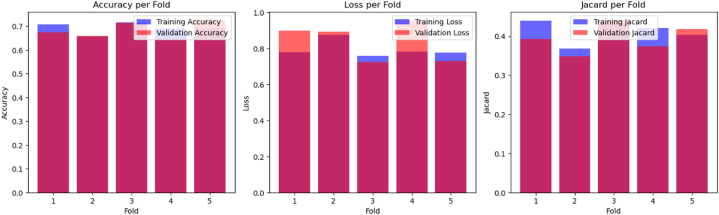
5-Fold cross-validation performance metrics.

**Fig. 13:**
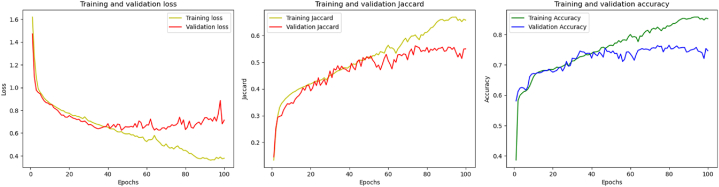
Model Loss, IoU, and Pixel Accuracy Plots of U-Net before deploying it with transfer learning.

A multiclass U-Net model was implemented for image segmentation using a 5-fold cross-validation strategy. The model was compiled with the Adam optimizer and categorical cross-entropy loss function and trained for 100 epochs per fold. The training procedure was verbose, with no data shuffling within epochs. Performance metrics for each fold's training and validation were recorded. The results, illustrated in Fig. 11, Fig. 12, show consistent improvement in both training and validation accuracy, a steady decrease in loss, and a progressive enhancement of the Jaccard index over epochs. These trends indicate effective learning, with validation performance closely following training performance, suggesting good generalization and minimal overfitting (see Fig. 13).

The U-Net model is trained on 48 × 48 pixel patches randomly extracted from pre-processed full images, including regions outside the field of view (FOV). This approach helps in distinguishing FOV borders and blood vessels. Training patches are obtained from DRIVE images without overlap or augmentation. Testing involves 20 DRIVE images, with FOV identified by masks, and performance is evaluated against a gold standard. To enhance performance, pixel probabilities from overlapping patches are averaged using a stride of 5 pixels. The results are benchmarked against recent methods based on accuracy scores in the DRIVE dataset. The performance of this neural network, tested on the DRIVE database, achieves the highest accuracy compared to other methods reported in the literature.

The proposed method also demonstrates one of the best performances on the STARE dataset. For this evaluation, the model was tested with modifications in code and methodology compared to the DRIVE dataset. The STARE database consists of 20 retinal fundus images, with manual segmentations provided by two observers, using one as the ground truth. Unlike DRIVE, STARE does not have a predefined train-test split; therefore, a leave-one-out cross-validation approach was employed, iterating across all 20 images.

During training, 9500 patches per image were used, with 90 % allocated for training and 10 % for validation. Patches were extracted similarly to DRIVE, including regions outside the FOV. Testing results were averaged over overlapping patches, and the FOV was determined through color thresholding due to the absence of masks in STARE. Performance results are summarized in Table 4, with accuracy as the key metric.

**Table 4:** Accuracy of different methods on the STARE.

Methods	Accuracy
[46]	95.1 % With Enhancement
[47]	97.35 %
[49]	96.80 %
[50]	94.8 %
[51]	98.13 %
Ours	98.15 %

The model exhibits high accuracy when segmenting individual areas, achieving approximately 0.98 accuracy for blood vessels and 0.983 for the optic disc and cup. However, the challenge of this study lies in simultaneously segmenting all regions, including the alpha and beta zones. Initial performance evaluation of the U-Net model yields the following metrics: model loss of 0.6367, pixel accuracy of 0.7466, Intersection-Over-Union (IoU) score of 0.5410, Dice Coefficient (F1 Score) of 0.6404, and Mean Intersection over Union (mIoU) of 0.3594. To address the need for further refinement, Transfer Learning is introduced. This approach utilizes pre-existing knowledge from related tasks to enhance the U-Net model's performance. By leveraging Transfer Learning, the model gains improved accuracy, better segmentation capabilities, and overall increased effectiveness in handling complex patterns within the data.

### 3.3 Evaluation of training and validation loss

**Fig. 14:**
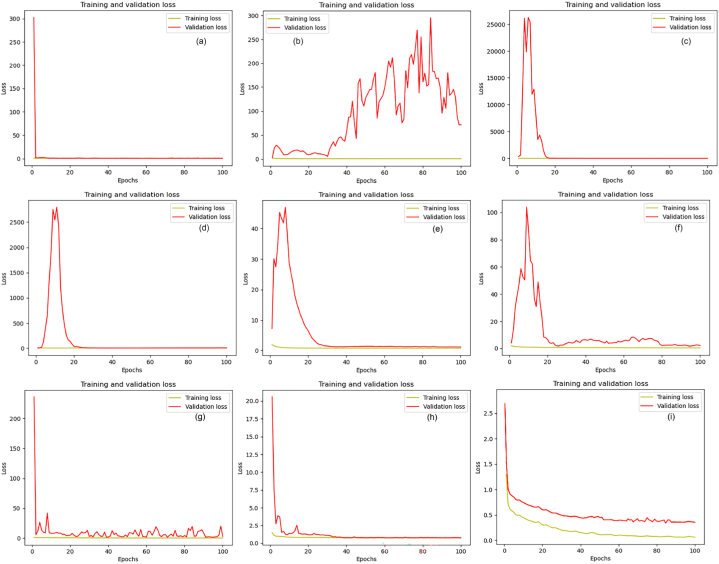
Loss plots: (a) DenseNet-121, (b) EfficientNetB0, (c) Inception-ResNet-v2, (d) Inceptionv3, (e) MobileNet, (f) SEResNet34, (g) SEResNeXt50, (h) VGG-19 and (i) proposed U-Net.

Various evaluation metrics were employed to assess both the proposed U-Net model and the transfer learning models. These metrics include model loss, Dice Coefficient (F1 Score), Intersection over Union (IoU), pixel accuracy, and Mean Intersection over Union (mIoU). Fig. 14a–i illustrate the loss curves during training and validation, offering insights into the model's performance over epochs.

The pre-trained models exhibited notable variations and distinct performance characteristics. In contrast, the proposed model, as shown in Fig. 14i, demonstrated more consistent performance. Notably, the proposed model outperformed the transfer learning models by achieving a lower loss. This indicates that the proposed model better optimized the model parameters, effectively minimizing the discrepancies between predicted and ground truth segmentations.

### 3.4 Analysis of Intersection-Over-Union (IoU)

**Fig. 15:**
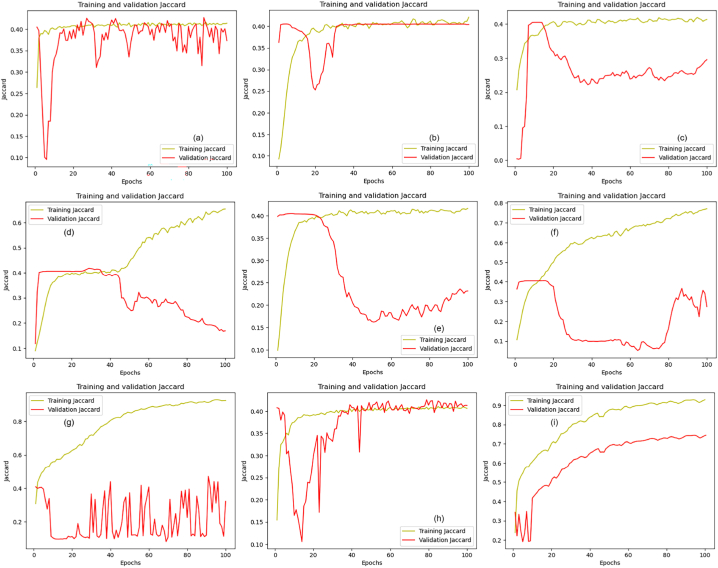
Jaccard index plots: (a) DenseNet-121, (b) EfficientNetB0, (c) Inception-ResNet-v2, (d) Inceptionv3, (e) MobileNet, (f) SEResNet34, (g) SEResNeXt50, (h) VGG-19 and (i) proposed U-Net.

Fig. 15a–i illustrate the Intersection over Union (IoU) curves for both the transfer learning models and the proposed model, tracked over training and validation phases. A close examination of these figures reveals that the performance of the pre-trained models is inconsistent, with noticeable variations in their IoU curves. In contrast, the graph presented in Fig. 15i demonstrates a more stable and consistent pattern. This consistency suggests that the proposed model is more robust and reliable compared to the transfer learning models. Moreover, the proposed model achieved the highest IoU score among all evaluated models, indicating superior capability in accurately segmenting objects in images. Overall, the proposed model not only shows better consistency but also surpasses the transfer learning models in accuracy, affirming its robustness and effectiveness for object segmentation.

### 3.5 Analysis of pixel accuracy

**Fig. 16:**
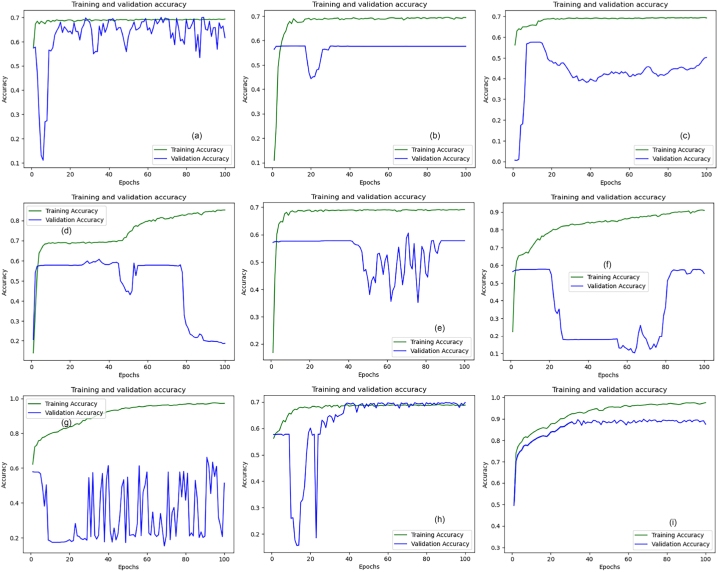
Pixel accuracy plots: (a) DenseNet-121, (b) EfficientNetB0, (c) Inception-ResNet-v2, (d) Inceptionv3, (e) MobileNet, (f) SEResNet34, (g) SEResNeXt50, (h) VGG-19 and (i) proposed U-Net.

Fig. 16a–i illustrate the pixel accuracy patterns observed throughout the training and validation phases for both the pre-trained models and the proposed model. Analysis of these figures reveals that the eight transfer learning models exhibit notably similar performance, with their pixel accuracy plots showing only minor variations. In contrast, the plot presented in Fig. 16i highlights the superior performance of the proposed model. It achieved the highest pixel accuracy among all the models evaluated, surpassing the transfer learning models. This indicates that the proposed model is more effective at correctly classifying pixels in the images, demonstrating its enhanced accuracy in segmentation tasks.

**Fig. 17:**
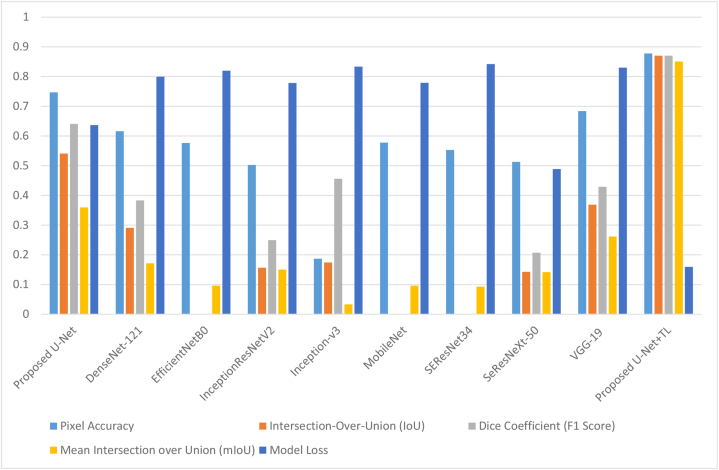
Pixel Accuracy, F1 Score, Model Loss, IoU Coefficient and mIoU Comparison Graph.

Fig. 17 presents a graphical comparison of the model loss, IoU, pixel accuracy, dice coefficient, and mean IoU. It is evident from Table 5 that the nine transfer learning models did not achieve alike results. The pre-trained models obtained the following model loss values: 0.799 for DenseNet-121, 0.819 for EfficientNetB0, 0.778 for InceptionResNetV2, 0.833 for Inception-v3, 0.779 for MobileNet, 0.842 for SEResNet34, 0.488 for SeResNeXt-50, and 0.829 for VGG-19. In contrast, the proposed model reached a model loss value of 0.1592. Based on the presented results, it can be deduced that the proposed model demonstrates superior performance compared to all the other pre-trained models, as it achieved the lowest loss value. Additionally, Fig. 17 shows that all eight pre-trained models reached identical outcomes. The pre-trained models accomplished the IoU values of 0.29 for DenseNet-121, 0.0008 for EfficientNetB0, 0.156 for InceptionResNetV2, 0.174 for Inception-v3, 0.0 for MobileNet, 0.0 for SEResNet34, 0.142 for SeResNeXt-50, and 0.368 for VGG-19. IoU calculates the overlap between the predicted and ground truth regions and is often used as a measure of segmentation quality. A higher IoU indicates better alignment between the predicted and true segmentations. The proposed U-Net achieves the highest IoU of 0.8704, indicating strong segmentation performance. Pixel accuracy measures the percentage of correctly classified pixels, where higher values indicate better accuracy in pixel-level classification. The proposed U-Net achieves the highest pixel accuracy of 0.8777, indicating its strong performance in correctly classifying individual pixels. The dice coefficient is another metric commonly used in image segmentation tasks. It quantifies the overlap between the predicted and true segmentations, providing a measure of similarity. Like IoU, a higher Dice coefficient signifies better segmentation performance. The proposed U-Net achieves a Dice coefficient of 0.8699, demonstrating strong performance. Furthermore, the mean Intersection over Union metric calculates the average IoU across all classes or regions of interest, providing a more comprehensive measure of the model's segmentation performance. The proposed U-Net achieves an mIoU of 0.8504, indicating its effectiveness in capturing similarities between predicted and true segmentations.

**Table 5:** Comparison Table of Pixel Accuracy, F1 Score, Model Loss, IoU Coefficient and mIoU.

Models	Pixel Accuracy	(IoU)	Dice Coefficient	(mIoU)	Model Loss
Proposed U-Net	0.746579064	0.541049508	0.640415223	0.359358503	0.63675
DenseNet-121	0.616284688	0.290706694	0.383257284	0.171642483	0.7994
EfficientNetB0	0.576722039	0.000814446	0.000690036	0.096423864	0.8198
InceptionResNetV2	0.502420213	0.156321598	0.249390466	0.150457479	0.7784
Inception-v3	0.186717139	0.174120113	0.456165653	0.033400765	0.8332
MobileNet	0.577840169	0	0	0.096306695	0.7792
SEResNet34	0.553046756	0	0	0.09261167	0.8421
SeResNeXt-50	0.512632582	0.142468698	0.206820095	0.141433241	0.4887
VGG-19	0.683692932	0.36875682	0.429162723	0.261745151	0.8299
Proposed U-Net + TL	0.877757874	0.870377352	0.869963776	0.850377352	0.1592

### 3.6 Comparative assessment of U-Net with transfer learning for multiregional image segmentation

The proposed model is evaluated against other models based on application, segmented areas, and data types, given the absence of similar comparisons. As highlighted in Table 6, the proposed model distinguishes itself with its complexity and ambition, featuring an ensemble of DenseNet-121, EfficientNetB0, InceptionResNetV2, Inception-v3, MobileNet, SEResNet34, SeResNeXt-50, VGG-19, and ResNet-34. In comparison, certain models, such as U-Net with ResNet-34, VGG16 for low-grade glioma segmentation, and 3D U-Net for ductal carcinoma classification, demonstrate effective results in specific tasks. However, these models are generally tailored to particular segmentation challenges. In contrast, the proposed model's ability to handle multiple segmented regions within fundus images represents a notable advantage. In summary, the proposed model offers a distinctive and comprehensive approach to segmentation, especially for complex fundus images. Although it may not outperform highly specialized models in every individual metric, given the varied data and applications discussed in Table 6, its broad applicability and competitive performance across multiple segmented regions position it as a promising candidate for diverse medical image segmentation tasks.

**Table 6:** Comparative analysis of U-Net with transfer learning models for medical image segmentation: Performance and insights.

**Ref.**	Used U-Net with PT Models	Segmented Area	Dataset	Input Image	Pixel Acc.	(IoU)	Dice Coeff.	(mIoU)	Model Loss
[52]	ResNet-34	Buildings	INRIA Aerial Image Labeling	Aerial	N/A	N/A	N/A	81.57	N/A
[53]	VGG19	Knee Menisci	SKI10 & 3D-FSE	MRI	N/A	N/A	0.833	N/A	N/A
[54]	ResNet-34	Three Retinal Layers	self-prepared	OCT	N/A	N/A	>0.9	N/A	N/A
[55]	Lightweight U-Net	Catheter	3 Dataset of Synthetic Fluoroscopic Images	X-ray	N/A	N/A	0.55	N/A	N/A
[56]	2.5D U-Net ResNet-34	Infant Brain Ventricles with Hydrocephalus	Adult Dataset	MRI	N/A	N/A	0.72	N/A	N/A
[57]	VGG16	Brain Tumor	Dr. Soetomo	MRI	CCR: 0.956	N/A	N/A	N/A	0.054
[58]	3D U-Net CNN	Pulmonary Nodules	LUNA16 & TIANCHI17	CT Scans	Acc.: 0.866	N/A	N/A	N/A	0.456
[59]	Residual	Psoriasis Lesion	self-prepared	Psoriasis Digital Images	N/A	0.901	0.948	N/A	N/A
[60]	FC-RNN	Breast and fibro glandular tissue	self-prepared	MRI	N/A	N/A	0.83	N/A	N/A
[61]	ResUnet MResUnet	Cross-Tissue/Organ	self-prepared	Ultrasound	N/A	0.607	0.746	N/A	N/A
[62]	VGG16	Low-Grade Gliomas	TCGA	MRI	N/A		0.996	N/A	0.06
[63]	VGG ResNet Res2Net	PPA	PALM	Fundus Image	N/A	0.703	0.81	N/A	N/A
[64]	VGG16	Ribs from Lung	LUS	Ultrasound	N/A	N/A	0.817	N/A	N/A
[65]	3D U-Net	Ductal Carcinoma Classification	Histopathological data of breasts	Microscopic Images	Acc.: 0.97	N/A		N/A	N/A
[66]	STL U-NET vanilla U-NET	Dynamic Speech	BRATS & AAPM	MRI & CT scan		N/A	0.79	N/A	N/A
[67]	Vgg19 Vgg16 Resnext50 DenseNet121 Resnet50	Breast Lesions	BUSI	Ultrasound	N/A	N/A	0.737	0.613	0.337
[68]	EfficientNetB2 ResNet34 ResNeXt50 InceptionV3	Prostate Adenocarcinoma	PANDA	Whole slide Images (WSI)	N/A	0.811	0.891	N/A	N/A
[69]	Inception V3 SeResNet50 VGG19 DenseNet121 InceptionResNetV2 EfficientNetB0	Gastrointestinal Tract	UW-GI TractSeg	MRI	N/A	0.768	0.604	N/A	0.418
**Proposed Model**	DenseNet-121 EfficientNetB0 InceptionResNetV2 nception-v3 MobileNet, SEResNet34 SeResNeXt-50 VGG-19 ResNet-34	Optic Disc, Optic Cup, Blood Vessel, PPA-Bata Zone and PPA-Bata Zone	HRF	Fundus Image	0.877	0.870	0.869	0.850	0.159

### 3.7 Explainability results

We deployed the proposed computational framework for segmentation, classification, and explainability of medical images using a range of deep learning models. Our analysis of explainability focused on evaluating model performance and interpretability through SHAP (SHapley Additive exPlanations) values, LIME (Local Interpretable Model-agnostic Explanations), and Grad-CAM (Gradient-weighted Class Activation Mapping) techniques.

The results of the explainability analysis demonstrated that our proposed U-Net model significantly surpassed pre-trained models—including DenseNet-121, EfficientNetB0, InceptionResNetV2, InceptionV3, MobileNet, SEResNet34, SeResNeXt-50, and VGG-19—in terms of Intersection over Union (IoU), pixel accuracy, and model loss. Specifically, the U-Net achieved the highest IoU of 0.8704, the highest pixel accuracy of 0.8777, and the lowest model loss of 0.1592, illustrating its superior capability in accurately segmenting objects within medical images.

Additionally, visual explanations generated via Grad-CAM indicated that the U-Net consistently focused on pertinent regions of the images, providing clearer and more interpretable saliency maps compared to transfer learning models. This finding underscores the robustness and reliability of our model for real-world applications.

While our study validates the effectiveness of the proposed model and provides valuable insights into the decision-making processes of deep learning models, it has limitations. The small dataset may restrict the generalizability of our findings, and the model's performance across different populations and imaging types remains uncertain. The complexity of deep learning models can also impede their clinical applicability. Assumptions of consistent data and disease patterns may not hold in real-world scenarios. Furthermore, limited data diversity and potential biases in data selection and labeling could impact validity. Future research should address these issues by employing larger datasets, enhancing model interpretability, and updating the model regularly.

### 3.8 Visual Analysis of segmented images

**Fig. 18:**
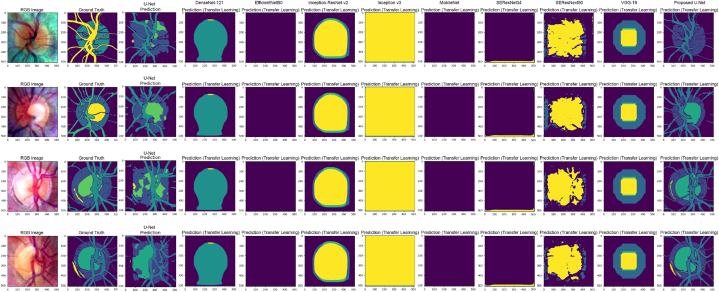
Visual analysis of the results.

Fig. 18 presents an illustrative depiction of the obtained results, including key components such as input images, corresponding ground truth data acquired through Run-Length Encoding (RLE), outputs from the U-Net model, and images generated by the proposed model. For clarity, a color-coded scheme is utilized within the figure: yellow represents the alpha zone, yellow-green denotes the beta zone, and medium-sea-green indicates the optic cup. The optic disc is marked with a dark shade of blue, while the intricate blood vessel network is depicted in dark cyan. This color-coded legend serves as a visual aid for the precise identification and differentiation of the ocular structures under analysis.

Upon examination, the images predicted by our proposed model closely align with the original ground truth, demonstrating the model's effectiveness in capturing segmentation patterns and identifying ocular structures. The congruence between the predicted images and the ground truth, facilitated by Run-Length Encoding, highlights the model's capability to accurately grasp features and perform segmentations. The integration of advanced segmentation techniques, such as the proposed model and Run-Length Encoding, is crucial for achieving precise delineation of ocular structures. This, in turn, enhances the accuracy and reliability of medical image analysis, underscoring its practical significance.

## 4 Conclusions

In conclusion, this paper presents a novel approach for precise optic nerve head segmentation in retinal fundus images. By integrating the advantages of training U-Nets from scratch with the benefits of transfer learning, our dual-U-Net architecture (BU-Net and MSU-Net) achieves superior accuracy in segmenting the optic disc and optic cup. Comprehensive image preprocessing and post-processing techniques ensure the generation of reliable binary masks, while the incorporation of a third U-Net trained on a diverse HRF dataset enhances the model's robustness. The inclusion of nine pre-trained CNNs further demonstrates the model's versatility and effectiveness. Our experiments with HRF images reveal substantial improvements in segmentation accuracy and robustness, establishing this as the first multi-class segmentation model specifically for the optic nerve head. The research addresses key aspects such as scalability, interoperability, and regulatory compliance, thereby providing a robust foundation for real-world clinical applications. Future work should focus on expanding the dataset, exploring multimodal data integration, and enhancing model interpretability to improve generalizability and clinical utility. Although significant progress has been made, it is essential to recognize potential limitations, including dataset size and diversity. Addressing these challenges will be crucial for advancing the model's contribution to the diagnosis and management of optic nerve diseases.

## Data availability statement

The High-Resolution Fundus (HRF) Image Database was used in this paper; however, it was modified and augmented with additional data by expert ophthalmologists to enrich the database and better suit the requirements of this work and future research.

## CRediT authorship contribution statement

**Najdavan A. Kako:** Writing – review & editing, Writing – original draft, Visualization, Validation, Software, Resources, Project administration, Methodology, Investigation, Funding acquisition, Formal analysis, Data curation, Conceptualization. **Adnan M. Abdulazeez:** Supervision, Investigation, Project administration. **Diler N. Abdulqader:** Resources, Data curation, Methodology.

## Declaration of competing interest

The authors declare that they have no known competing financial interests or personal relationships that could have appeared to influence the work reported in this paper.
